# Increased Frequency of Rheumatoid Arthritis and Allergic Rhinitis among Pesticide Sprayers and Associations with Pesticide Use

**DOI:** 10.3390/ijerph14080865

**Published:** 2017-08-01

**Authors:** Michalis Koureas, George Rachiotis, Andreas Tsakalof, Christos Hadjichristodoulou

**Affiliations:** Department of Hygiene and Epidemiology, Faculty of Medicine, University of Thessaly, 22 Papakyriazi Street, 41222 Larissa, Greece; mkoureas@med.uth.gr (M.K.); grachiotis@gmail.com (G.R.); atsakal@med.uth.gr (A.T.)

**Keywords:** pesticides, rheumatoid arthritis, allergic rhinitis, sprayers

## Abstract

*Objective*: The aim of this study was to identify diseases linked with the pesticide sprayer occupation and explore possible associations with exposure history data. *Methods*: A cross sectional study was conducted among pesticide sprayers (*n* = 80) and the general population (*n* = 90) in Thessaly (Greece). Medical history, demographic characteristics and detailed exposure history were recorded by conducting personal interviews. Lifetime exposure indicators were calculated for several pesticide chemical subclasses. Moreover, organophosphate metabolite levels were quantified in urine samples of all participants by using gas chromatography-mass spectrometry (GC-MS). Multinomial analysis was used to determine associations between occupational pesticide exposure and diseases or disorders. *Results*: In the pesticide sprayers group, significantly higher frequencies for rheumatoid arthritis (RA) and allergic rhinitis were observed compared with the control group (*p* = 0.002 and *p* = 0.024 respectively). Within the pesticide sprayers group, high lifetime pesticide exposure was associated with increased risk for reporting RA (OR: 43.07 95% CI: 3.09–600.67) and allergic rhinitis (OR: 9.72 95% CI: 2.31–40.89), compared with low pesticide exposure. Exposure to organophsphate, guanidine and quinone pesticides were associated with RA while organophosphates, pyrethroids and paraquat were associated with allergic rhinitis. Despite the higher levels of certain pesticide metabolites observed among participants with rheumatoid arthritis, the differences were not statistically significant. One metabolite (diethylthiophosphate) was found to be significantly increased in allergic rhinitis cases (*p* = 0.037). *Conclusions*: The results from the current study suggest a possible association of occupational pesticide exposure with RA and allergic rhinitis that should be further investigated.

## 1. Introduction

The use of pesticides in agriculture guarantees the protection of crops from undesirable pests and has beneficial effects on agricultural production. However, there is a huge body of scientific evidence concerning the adverse health effects of pesticide exposure although sometimes findings are conflicting. Exposure to pesticides has been associated with a variety of health effects including neurodegenerative diseases, cancer and neurobehavioral disorders [1,2,3,4]. Also, toxicological data support the genotoxic [5] and immunotoxic [6] effects of pesticides, while numerous active substances used as pesticides are considered endocrine disrupting chemicals (EDCs) [7].

Although the impact of pesticides on human health has been extensively studied, assessing exposure to pesticides and linking it with health outcomes remains a challenge for the scientific community. Unlike other health hazards, pesticides consist of a wide range of chemicals ranging from inorganic substances to modern synthetic compounds. Also, the array of pesticides used in agriculture is constantly changing with newer substances replacing older more toxic or more persistent ones. The diversity of the active substances that constitute the group “pesticides” highlights the need for isolating the effects of exposure to specific substances or chemical classes in human studies. This is more important for relatively newly developed pesticides since not much is known about their potential health effects, especially as a result of long term exposure [8].

Pesticide sprayers are by definition a population group with increased exposure to insecticides, herbicides and fungicides. In a previous study, we demonstrated that pesticide sprayers had significantly higher levels of urinary metabolites of organophosphate pesticides and that these biomarkers of exposure are affected by the use of personal protective equipment (PPE) and the application of hygiene practices during loading and applying pesticides [9]. Moreover, we observed increased levels of oxidative DNA damage in whole blood samples collected from pesticide sprayers [10]. In the current study, we present the results of a questionnaire based survey on pesticide sprayers and non-exposed controls. The objective of the study was to identify differences in frequencies of disease occurrence between population groups but also to identify specific pesticides or pesticide chemical classes that are related to health outcomes.

## 2. Materials and Methods

### 2.1. Study Area and Study Population

The characteristics of the area of the study and the study population have been reported in a previous publication [9]. Briefly, the study was conducted in an agricultural area of central Greece, with intensive agriculture and high pesticide use. A list of farm owners was obtained through the local citizen’s service center. In the particular agricultural community, it is common practice for farm owners to apply pesticides themselves rather than hiring others to perform the task. Out of approximately 200 farm owners contacted, 115 fulfilled the criteria and 80 participated to the personal interviews (response rate: 69.56%).

At the same time, 90 male residents from the general population of the city of Larisa (without occupational exposure) were recruited and used as a control group. Urban residents were mainly blood donors who were contacted during voluntary blood donations, and employees of the University of Thessaly. We also recruited volunteers from the nursing home in the city of Larissa; in order to match for age the participants in the exposed group were more than 65 years old. The protocol of the study has been approved by the General Assembly of the Medical Faculty, School of Health Science at the University of Thessaly, Greece. All participants were informed about the objectives of the study and signed a consent form. In the same populations (pesticide sprayers group) we have observed increased levels of urinary organophosphate metabolites and oxidative DNA damage in a previous study [9,10].

### 2.2. Questionnaires and Calculation of Exposure Indicators

Sociodemographic characteristics were recorded from all participants by conducting personal interviews. Details on smoking and alcohol consumption were also recorded. For the pesticide sprayers group, a detailed exposure questionnaire was completed by conducting personal interviews with each of the participants. Lifetime pesticide exposure was asessed by asking the participants to report the total years of pesticide usage, the area and type of crop treated, the commercial names of the pesticides they have used and the frequency of application (applications per year) per pesticide. By using information derived from questionnaires, exposure indicators were calculated.

The following chronic (lifetime) exposure indicators were used:

Pesticide exposure Indicator 1 = {*Number of Applications per year*} × {*total years applying pesticides*}

Pesticide exposure Indicator 2 = {*Number of Applications per year*} × {*total years applying pesticides*} × {*area of application*}

Indicator 1 corresponds to the total number of pesticide applications throughout the lifespan and Indicator 2 corresponds to the total area sprayed throughout the lifespan. Indicators of exposure were calculated for total pesticide usage, insecticides, fungicides and herbicides, while indicators of exposure to chemical subclasses of pesticides (e.g., organophosphates, pyrethroids, neonicotinoids, etc.) were also calculated. Deatails on the use of PPE were also recorded. Moreover, detailed data on medical history were collected by using a separate questionnaire which contained an extensive list of diseases and participants were asked if they have ever been diagnosed with any of them.

### 2.3. Laboratory Analysis

Spot urine samples were collected from each participant in the study. Samples were transferred at 4 °C to the Laboratory of Hygiene and Epidemiology, University of Thessaly, Greece and were frozen and stored at −20 °C until there were tested. For analysis, the samples were shipped to the Laboratory of Toxicology and Forensic Chemistry, Department of Morphology, Faculty of Medicine University of Crete, Greece. In the urine samples, the concentration of four non-specific metabolites of OPs was determined. A detailed description of the analytical method used can be found in a previous publication [9]. The limits of quantitation (LOQ) were 0.2 ng/mL for dimethylphosphate (DMP), 0.3 ng/mL for diethylphosphate (DEP), 0.1 ng/mL for diethylthiophosphate (DETP) and 0.1 ng/mL for diethyldithiophosphate (DEDTP). The selected metabolites reflect the exposure to the corresponding parent compounds that are still being used in the study area, namely chlorpyriphos, phosmet and dimethoate.

### 2.4. Statistical Analysis

Statistical analysis was carried out by using the Statistical Package for Social Sciences (SPSS) version 15.0 (SPSS Inc., Chicago, IL, USA). The non-parametric Mann–Whitney test was used to examine differences between quantitative variables. Multinomial logistic regression was used to estimate the association between exposure groups (sprayers vs. controls) and disease status. Age, smoking, and alcohol consumption were included in the model. Additional statistical analysis was conducted within the homogeneous exposure group of pesticide sprayers. For the diseases found to have statistically significant associations with occupational exposure, exposure indicators were compared between self-reported cases and non-cases using Mann–Whitney test. Consequently, for the pesticide classes that had significantly elevated exposure indicators in cases, these indicators were transformed to categorical variables using ROC curve analysis to determine cutoff values. Multivariate analysis was then performed to calculate the corresponding odds ratios (OR) and their 95% confidence intervals (95% CI). For the abovementioned diseases, pesticide metabolite levels were compared between cases and healthy individuals by using the Mann–Whitney test. A *p*-value < 0.05 was considered statistically significant. 

## 3. Results

Mean age was 48 (SD:12) for sprayers and 47 (SD:12) for the control group, and regular smoking was reported by 35.0% of sprayers and 45.6% of the control group. The groups differed in education status with 52.2%, of the controls reporting higher education compared with 17.5% of the sprayers. Six cases of RA were reported in the pesticide sprayers group while no cases were recorded in the control group. Multinomial logistic regression analysis including as confounders age, smoking and alcohol consumption revealed a statistically significant association between pesticide sprayer occupation and RA (OR = undefined [d = zero], *p* = 0.002). Also, pesticide sprayers were found to have increased odds for reporting allergic rhinitis, compared to the non-exposed controls (OR: 2.90, 95 CI: 1.15–7.29, *p* = 0.024). No statistically significant diference was found among the two groups of participants (sprayers and controls) rearding all the other diseases included in the questionnaire.

For RA and allergic rhinitis, additional analysis was performed within the pesticide sprayers group. Mann-Whitney test revealed statistically significant differences in pesticide exposure indicators between self-reported cases and the rest of the sprayers. Specifically, RA cases had elevated values in the following lifetime exposure indicators: (i) total pesticide exposure (*p* = 0.026); (ii) total insecticide exposure (*p* = 0.036); (iii) total fungicide exposure (*p* = 0.013); (iv) organophosphates (*p* = 0.012); (v) guanidine (*p* = 0.06); and (vi) quinones (*p* = 0.024). Allergic rhinitis cases had higher exposure indicators for (i) total pesticide exposure (*p* = 0.013); (ii) total insecticide exposure (*p* = 0.006); (iii) total herbicide exposure (*p* = 0.018); (iv) organophosphates (*p* = 0.014); (v) pyrethroids (*p* = 0.016) and paraquat (*p* = 0.013). These statistically significant associations are prone to confounding effects of other variables, and most notably age, which is strongly related to pesticide exposure indicators. In order to address this issue and control for confounders, exposure indicators were transformed to categorical variables and multivariate logistic models were run. Cutoff values were determined by ROC curve analysis. Table 1 and Table 2 present the results of the multivariate analysis. All associations identified by Mann–Whitney test remained significant in the multivariate analysis.

Urinary dialkyl phoshphate levels were compared between self-reported cases of RA and allergic rhinitis and individuals without the disease. The results of the comparison are presented in Table 3. Certain metabolites were elevated in self reported cases but in most cases differences were not statistically significant. Allergic rhinitis cases had statistically significant higher levels of diethylthiophosphate.

## 4. Discussion

In this study, we observed a surprisingly high frequency (7.5%) of self-reported rheumatoid arthritis among pesticide sprayers. Taking into account the fact that the prevalence of rheumatoid arthritis ranges between 0.5–1% [11], and in males even less, this finding suggests a potential relationship with pesticide exposure. Having recorded the complete exposure history of the participants, we were able to search for more evidence that could support the hypothesis driven from the analysis of cross sectional data. The analysis of exposure indicators within the group of pesticide sprayers showed that self-reported cases had increased lifetime exposure indices. Although the sample size was small, the differences were statistically significant for some pesticide indicators, including total pesticide exposure indicator. It should be noted that the calculated ORs in the multivariate analysis do not reflect a realistic picture of the magnitude of risk because of the small number of cases. The analysis, however, provides evidence that justifies further investigation. Moreover, in our analysis it was observed that the size of the area of application did not seem to increase the risk of disease. On the contrary, when the area of pesticide application was not incorporated to the exposure indicator, the associations of these indicators with RA and allergic rhinitis were notably stronger. Although there is no ready explanation for this, we assume that the larger area of application implies an increased probability of applying more efficient protective measures, leading to exposure mitigation. Our analysis showed that pesticide sprayers who possessed a tractor with a closed cabin cultivate significantly larger areas compared with those who did not (median 7ha [IQR: 4–10] vs. 3ha [IQR: 2.4–5]).

Professional involvement in agriculture has been associated with autoimmune rheumatoid arthritis in different populations by using different study designs and in some of them the relationship was atributed to pesticides exposure [12,13,14,15]. The mechanisms by which pesticides can cause rheumatoid arthritis vary and few animal studies have been conducted. It has been suggested that pesticides may impact differentiation and regulation of adaptive and innate immune responses [16]. Pesticide exposure may be related to modification of Th1/Th2 cytokine profiles, elevated pro inflammatory cytokine levels and increased autoantibodies [17,18]. Allergic rhinitis was also more frequently reported in pesticide sprayers and individuals who reported having allergic rhinitis had significantly higher exposure indicators. Specific classes of pesticides were identified which included organophosphates, pyrethroids and the herbicide paraquat. Exposure to paraquat has been associated with allergic rhinitis in a previous study from the Greek Island of Crete [19], while analysis of data from the US agricultural health study showed a consistent association with (among others) the organophosphate Diazinon [20]. Organophosohorus insecticides inhibit acetylcholinesterase activity leading to acetylocholine accumulation. Cholinergic stimulation of the nasal mucosa results in increased nasal lavage secretions and subjective nasal congestion. It has been hypothesized that continuous organophosphate exposure may lead to exaggerated nasal glandular response [20]. 

Although pesticide exposure history and particularly exposure to organophosphorus insecticides was found to have a statistical correlation with disease occurrence, organophosphate metabolite measurements did not have solid associations with diseases. Dialkyl phosphate metabolites were present in the vast majority of both sprayers and non-occupationally exposed participants, reflecting not only occupational but also dietary exposures [9]. Specific metabolites were found to be elevated in self-reported cases but statistically significant higher levels of only diethylthiophosphate were found only in allergic rhinitis cases. The absence of statistically significant associations can be attributed to the small number of cases but can also be explained by the fact that these substances reflect short term exposure and are not the most appropriate way of studying long term effects, especially in a cross sectional design [21].

The findings reported should be carefully interpreted, given the limitations that may exist in the specific study. Some key constraints are: (i) that the findings are based on the analysis of cross-sectional data; (ii) the characterization of a participant as a case for a disease was based on self-report; (iii) exposure metrics were derived retrospectively from personal interviews and are prone to recall bias; (iv) existing associations with other diseases/disorders may have not been revealed due to the small sample size. Moreover, pesticide sprayers were recruited from a specific agricultural area, and therefore the observed differences in disease frequencies between sprayers and controls can be due to a variety of other factors and environmental exposures (such as allergens) prevalent in the study area. However, RA and allergic rhinitis showed significant associations with pesticide exposure history within the pesticide sprayers group, a finding than could not have been biased by factors related to the study area.

The purpose of this study was not to document causal associations of pesticide exposures with specific diseases, but to examine potential relationships between a wide range of exposures and diseases and generate hypotheses. Under this framework, some interesting associations were revealed. Guanidine and quinone fungicides showed a strong statistical relationship with RA, and pyrethroids were associated with rhinitis. To our knowledge this is the first time that such associations are reported and further investigation is needed to examine a possible causal relationship. 

## 5. Conclusions

Pesticide sprayers were found to have increased frequencies of RA and allergic rhinitis compared to non-exposed controls. Additionally, our analysis showed that lifetime exposure indicators for organophsphate, guanidine and quinone pesticides were associated with RA, while organophosphates, pyrethroids and paraquat were associated with allergic rhinitis. These findings must be interpreted with caution due to the limitations of the present study. However, associations with some specific chemical classes (guanidines, quinones) are reported for the first time and thus further research on the possible relationship is needed.

## Figures and Tables

**Table 1:** Multivariate logistic regression to determine association between pesticide exposure indicator 1, rheumatoid arthritis and allergic rhinitis ^1^.

Pesticide Exposure Indicator 1 (High vs. Low) ^2^	RA (+) vs. RA (−)	Allergic Rhinitis (+) vs. Allergic Rhinitis (−)
	Odds Ratio (95% CI)	Odds Ratio (95% CI)
**Aggregate Exposure Indicators**		
Total pesticide exposure	**43.07 (3.09–600.67)**	**9.72 (2.31–40.89)**
Insecticides	**15.29 (1.24–189.02)**	**14.59 (2.86–74.36)**
Herbicides	5.01 (0.51–49.33)	**12.43 (1.45–106.59)**
Fungicides	**14.39 (1.38–150.37)**	**3.89 (1.07–14.13)**
**Indicators of Exposure for Pesticide Subclasses**		
Organophosphates	**34.27 (2.49–471.73)**	**19.69 (2.84–136.5)**
Pyrethroids	6.40 (0.42–96.97)	**4.73 (1.30–17.19)**
Paraquat	2.79 (0.38–20.88)	**8.03 (1.76–43.67)**
Guanidines	**77.52 (1.50–4005.80)**	1.34 (0.32–5.73)
Quinones	**18.72 (1.79–195.75)**	1.26 (0.34–4.65)

^1^ Adjusted for age, smoking, alcohol consumption and use of a tractor equipped with a closed cabin. ^2^ cutoff values determined by Receiver Operating Characteristic (ROC) curve. Statistically significant associations are highlighted with bold.

**Table 2:** Multivariate logistic regression to determine association between pesticide exposure indicator 2, rheumatoid arthritis and allergic rhinitis ^1^.

Pesticide Exposure Indicator 2 (High vs. Low) ^2^	RA (+) vs. RA (−)	Allergic Rhinitis (+) vs. Allergic Rhinitis (−)
	Odds Ratio (95% CI)	Odds Ratio (95% CI)
**Aggregate Exposure Indicators**		
Total pesticide exposure	3.90 (0.49–31.33)	2.58 (0.75–8.88)
Insecticides	2.82 (0.41–19.54)	3.11 (0.93–10.34)
Herbicides	3.51 (0.45–27.20)	3.15 (0.93–10.66)
Fungicides	5.85 (0.82–42.04)	2.41 (0.71–8.32)
**Indicators of Exposure for Pesticide Subclasses**		
Organophosphates	**6.47 (1.00–45.43)**	**3.33 (1.00–11.04)**
Pyrethroids	5.65 (0.39–81.82)	**6.92 (1.70–28.15)**
Paraquat	0.69 (0.094–5.03)	**9.10 (1.70–48.54)**
Guanidines	**16.18 (1.58–165.97)**	1.69 (0.48–5.97)
Quinones	**undefined ^3^**	1.67 (0.45–6.28)

^1^ Adjusted for age, smoking and alcohol consumption and use of a tractor equipped with a closed cabin. ^2^ cutoff values determined by ROC curve. ^3^ There were no rheumatoid arthritis (RA) cases in the low exposure group. Statistically significant associations are highlighted with bold.

**Table 3:** Levels of urinary organophosphate metabolites (μg/g creatinine) according to disease status.

	*n*	Median (IQR)	*p*-Value
**Dimethyl phosphate**			
RA (+)	6	6.58 (0.84–12.63)	1.000
RA (−)	164	3.63 (1.70–9.39)	
Allergic rhinitis (+)	25	3.63 (2.12–9.09)	0.808
Allergic rhinitis (−)	145	3.63 (1.54–10–13)	
**Diethyl phospate**			
RA (+)	6	4.28 (3.01–8.09)	0.755
RA (−)	164	4.54 (2.31–9.07)	
Allergic rhinitis (+)	25	6.47 (2.43–9.94)	0.468
Allergic rhinitis (−)	145	4.37 (2.45–8.60)	
**Diethyl thiophospate**			
RA (+)	6	2.47 (1.88–3.80)	0.675
RA (−)	164	2.36 (0.84–7.36)	
Allergic rhinitis (+)	25	**4.06 (2.35–7.66)**	**0.037**
Allergic rhinitis (−)	145	1.97 (0.82–6.86)	
**Diethyl dithiophospate**			
RA (+)	6	0.66 (0.47–3.16)	0.695
RA (−)	164	0.60 (0.34–1.17)	
Allergic rhinitis (+)	25	0.85 (0.47–1.17)	0.252
Allergic rhinitis (−)	145	0.59 (0.34–1.17)	
**Total dialkylphosphates**			
RA (+)	6	16.78 (5.79–56.55)	0.894
RA (−)	164	15.76 (7.94–29.06)	
Allergic rhinitis (+)	25	16.48 (12.45–26.21)	0.325
Allergic rhinitis (−)	145	15.58 (7.56–30.35)	

Statistically significant associations are highlighted with bold.
